# Beyond delabeling: long-term penicillin use and avoidance following negative penicillin challenge in children

**DOI:** 10.3389/fped.2026.1983812

**Published:** 2026-10-14

**Authors:** Vered Schichter-Konfino, Noam Sharter Even Tsur, Nir Vered, Adi Klein, Meital Almog, Eias Kasem

**Affiliations:** 1 Pediatric Allergy and Immunology Unit, Hillel Yaffe Medical Center, Hadera, Israel; 2 Bruce Rappaport Faculty of Medicine, Technion-Israel Institute of Technology, Haifa, Israel; 3 Pediatric Department, Meir Medical Center, Kfar Saba, Israel; 4 Pediatric Department, Hillel Yaffe Medical Center, Hadera, Israel; 5 Pediatric Allergy and Immunology Unit, Bnai Zion Medical Center, Haifa, Israel; 6 Clalit Health Services, Sharon-Shomron County, Netanya, Israel

**Keywords:** allergic reaction, antimicrobial stewardship, drug provocation testing, penicillin allergy, penicillin delabeling

## Abstract

**Background:**

Penicillin allergy labels are frequently reported in children and contribute to avoidance of first-line antibiotic therapy. In appropriately selected pediatric patients, direct penicillin challenge is an increasingly used strategy for allergy evaluation and delabeling. However, whether successful delabeling translates into subsequent penicillin use in routine clinical practice remains poorly understood.

**Purpose:**

This study evaluated long-term antibiotic use after successful penicillin allergy delabeling in children, focusing on subsequent penicillin reuse, continued avoidance, and factors associated with subsequent reuse.

**Methods:**

We conducted a retrospective cohort study of 305 children referred for penicillin allergy evaluation at the Hillel Yaffe Pediatric Allergy Clinic between 2019 and 2023. Patients underwent risk-stratified skin testing and/or direct oral challenge according to institutional protocol, followed by a 5-day home treatment when indicated. Long-term outcomes, including subsequent penicillin reuse and any adverse reactions following challenge, were assessed via structured telephone interviews with caregivers.

**Results:**

The median age at index reaction was 4 years; reactions were predominantly nonimmediate (59.6%) and cutaneous (98.0%). Oral provocation testing was performed in 93.8% of patients and was well tolerated in 98.6%. Full tolerance, defined as negative clinic challenge and negative 5-day home treatment, was documented in 79.7% of the cohort. Among fully tolerant children with follow-up, 63.7% subsequently reused penicillin, whereas 36.3% had no documented subsequent use. No subsequent allergic reactions were reported among children who reused penicillin. Younger age and shorter time since provocation were independently associated with greater likelihood of subsequent penicillin reuse.

**Conclusion:**

Direct oral challenge was safe and successfully established penicillin tolerance in most children. However, successful delabeling did not consistently translate into subsequent penicillin use, highlighting a clinically important gap between diagnostic delabeling and its real-world implementation. Improved post-delabeling communication, documentation, and integration into antimicrobial stewardship may help maximize the clinical benefit of penicillin allergy delabeling.

## 1 Introduction

Penicillin allergy labels are common in childhood and can substantially influence antibiotic prescribing throughout subsequent years. Although many children are labeled as allergic after suspected reactions to antibiotics, most reported reactions do not represent persistent immune-mediated allergy. Inaccurate penicillin allergy labels may lead to avoidance of first-line antibiotic and increased use of broader-spectrum alternatives, with implications for antimicrobial stewardship and healthcare utilization. These consequences are particularly relevant in children, because an allergy label acquired early in life may influence antibiotic selection repeatedly throughout childhood (1–4).

Drug hypersensitivity reactions in children are heterogeneous and vary according to the drug involved, clinical phenotype, timing, and underlying mechanism. β-lactam antibiotics are among the most frequently implicated drug classes (1, 5). Most suspected reactions are mild and predominantly cutaneous, including maculopapular exanthems and urticaria, whereas severe cutaneous adverse reactions (SCAR) such as Stevens-Johnson syndrome, toxic epidermal necrolysis, drug reaction with eosinophilia and systemic symptoms, and acute generalized exanthematous pustulosis are rare (1, 6). Many childhood rashes attributed to antibiotics occur in the setting of concurrent viral infections and do not represent persistent drug hypersensitivity (7, 8). Consequently, a reported history of penicillin allergy does not necessarily indicate clinically relevant, persistent penicillin hypersensitivity.

Penicillin is among the most commonly reported drug allergies in childhood, yet formal evaluation demonstrates that most children with a reported penicillin allergy can tolerate penicillin subsequently (7, 9, 10). A systematic review of pediatric penicillin allergy testing found that evaluation and subsequent removal of an inaccurate allergy label are generally associated with favorable clinical outcomes (10). Direct oral challenge has therefore become an important component of diagnostic evaluation, particularly for children with low-risk histories, while skin prick and intradermal testing remain useful in selected patients with histories suggestive of immediate hypersensitivity (11–15). Recent evidence supports the safety of direct challenge, with severe reactions being rare, and pediatric studies have demonstrated that risk-stratified direct oral challenge can be performed safely and effectively. A systematic review and meta-analysis of 56 studies including 9,225 participants found an overall reaction frequency of 3.5% during direct penicillin challenges, with severe reactions being rare (9). Pediatric studies have similarly demonstrated that direct oral challenges can be performed safely and effectively in appropriately selected children (12, 14, 16).

The increasing adoption of risk-based approaches has facilitated penicillin allergy delabeling beyond traditional specialist allergy settings. Clinical decision tools such as PEN-FAST have been evaluated for risk stratification (12), while prospective and real-world studies have demonstrated the feasibility of direct oral challenges and delabeling in pediatric emergency departments, hospitals, primary care settings, and allergy clinics (13–19). These approaches may improve access to allergy evaluation and allow delabeling to become an integral component of antimicrobial stewardship. However, successful delabeling should not be considered solely as a negative challenge or removal of the allergy label. Its ultimate clinical benefit depends on whether the previously avoided antibiotic is subsequently used when clinically indicated.

Although short-term outcomes following penicillin allergy delabeling are generally favorable, evidence regarding long-term real-world outcomes remains limited. Some patients continue to avoid penicillin despite successful evaluation and removal of the allergy label (20). In children, a systematic review found generally sustained tolerance following negative penicillin allergy evaluation, including studies with follow-up periods extending several years (10). Nevertheless, persistent avoidance of the delabeled antibiotic remains an important concern. A recent 5-year follow-up study found that most delabeled children subsequently reused the previously suspected antibiotic, but a substantial proportion did not (21). These findings suggest that successful diagnostic evaluation does not necessarily translate into changes in real-world prescribing or antibiotic use.

Several barriers may therefore limit the long-term impact of successful delabeling. Communication between allergy specialists, primary care clinicians, families, and other healthcare providers may influence whether a negative evaluation is recognized and acted upon (22). Even when delabeling has been successfully completed, clinicians or families may remain reluctant to use the previously suspected antibiotic because of uncertainty regarding the original reaction, incomplete communication of the negative challenge result, or concern about a recurrent reaction. Conversely, studies conducted in pediatric primary care and emergency settings demonstrate that structured communication and implementation strategies can facilitate the uptake of delabeling and subsequent use of first-line antibiotics (16–18).

Despite growing evidence supporting pediatric penicillin allergy delabeling, important gaps remain regarding its long-term clinical impact. In particular, limited data are available on actual penicillin utilization after documented tolerance, the extent to which children continue to avoid penicillin, and factors associated with subsequent reuse or continued avoidance. Furthermore, the relationship between successful allergy evaluation and subsequent antibiotic prescribing in real-world clinical practice remains incompletely characterized. Understanding these outcomes is important because the benefits of delabeling extend beyond the immediate diagnostic encounter and ultimately depend on sustained changes in antibiotic selection.

Therefore, this study aimed to evaluate long-term antibiotic utilization following penicillin allergy evaluation and successful delabeling in children, with particular emphasis on subsequent penicillin reuse and continued avoidance.

## 2 Methods

### 2.1 Subjects and measurements

We conducted a retrospective cohort study of pediatric patients referred for penicillin allergy evaluation at the Pediatric Allergy Clinic of Hillel Yaffe Medical Center between January 2019 and December 2023. The clinic serves as a tertiary referral center for drug allergy assessment.

All children underwent a clinical assessment of the reported index reaction, including the timing of symptom onset, clinical manifestations, severity, and characteristics of the reaction. Children with a history suggestive of severe cutaneous adverse drug reactions (SCAR) or other contraindications to drug challenge were excluded from challenge testing. The diagnostic approach was determined according to the clinical characteristics and risk profile of the reported reaction. Skin testing (skin prick and/or intradermal testing) was performed preferentially in children with significant urticarial index reactions. Skin testing was accordingly omitted in many children with a simple, non-severe rash, consistent with guideline recommendations that skin tests may be skipped before drug provocation testing in children with a benign non-immediate reaction to beta-lactams (23, 24).

Oral challenge was conducted under medical supervision as a two-step graded oral challenge to a target dose of 50 mg/kg (maximum 500 mg): initially 10% of the target dose was administered, followed by a 30-minute observation period; in the absence of an allergic reaction, the remaining 90% was administered, followed by an additional 2-hour observation period. Challenges were deferred in the presence of acute illness or uncontrolled asthma. Reactions were classified according to their chronology and clinical phenotype. Immediate reactions were defined as reactions occurring within 1–6 h after drug administration, whereas reactions occurring later were classified as nonimmediate reactions, according to the EAACI terminology (25). Patients who tolerated the supervised challenge without an immediate reaction were instructed to continue penicillin at home for 5 days at the age- and weight-appropriate therapeutic daily dose, in order to identify possible delayed reactions not apparent during the supervised challenge.

Clinical data were extracted from electronic medical records (EMR) and included demographic characteristics, the indication for the original penicillin prescription, index reaction features, skin testing results when performed, and challenge outcomes. Long-term follow-up data were obtained through structured telephone interviews with caregivers and included subsequent antibiotic use, penicillin reuse or avoidance, and any adverse reactions following challenge.

For the purposes of this study, full tolerance was defined as a negative supervised oral challenge followed, when applicable, by completion of the 5-day home treatment without a clinically significant reaction.

The primary outcome was the occurrence of an immediate or nonimmediate allergic reaction during the penicillin challenge. Secondary outcomes included penicillin use during follow-up.

### 2.2 Statistical analysis

Statistical analyses were performed using IBM SPSS Statistics version 31 (IBM Corp., Armonk, NY, USA). Continuous variables were presented as means with standard deviations for normally distributed data, and as medians (Me) with interquartile ranges (IQR) for non-normally distributed data, as assessed by the Kolmogorov–Smirnov test. Categorical variables were summarized as frequencies and percentages. Continuous variables were compared using the Mann–Whitney U test, and categorical variables were compared using Pearson's chi-square (*χ*^2^) test or Fisher's exact test, as appropriate. All statistical tests were two-sided, and a *p*-value of <0.05 was considered statistically significant.

An exploratory multivariable logistic regression model was constructed to evaluate associations between selected clinical variables and challenge positivity. The model included age, sex, time to reaction onset, urticaria, and time from index reaction to challenge. Complete-case analysis included 255 patients and 15 positive events, corresponding to 3 events per predictor variable. Given the limited number of events, adjusted estimates were considered exploratory and hypothesis-generating.

## 3 Results

A total of 305 pediatric patients referred between 2019 and 2023 for suspected penicillin allergy were evaluated. Baseline characteristics and index reaction features are described in Table 1. The patient flow through diagnostic evaluation, supervised oral challenge, 5-day home treatment, and long-term follow-up is presented in Figure 1. The median age at the time of index reaction was 4 years, with a balanced sex distribution (50.8% male). The most common indication for penicillin prescription was acute otitis media (32.5%), followed by streptococcal pharyngitis (18.0%), pneumonia (11.1%), non-specific pharyngitis (10.8%), and additional indications including sinusitis, infected wounds, and febrile illnesses (3.3%); the underlying diagnosis was unclear in 23.3% of cases.

**Table 1:** Baseline characteristics and index reaction features (*n* = 305).

Variables	Values
Demographic parameters	
Age (years), Me (IQR)	4.0 (2–7)
Gender, Male, *n* (%)	155 (50.8)
Indication for penicillin use, *n* (%)	
Acute otitis media	99 (32.5)
Streptococcal pharyngitis	55 (18.0)
Non-specific pharyngitis	33 (10.8)
Pneumonia	34 (11.1)
Other (sinusitis, fever, surgical procedure, infected wounds)	10 (3.3)
Diagnosis unclear	71 (23.3)
Time to reaction onset (days), Me (IQR)	2.0 (1–5)
Immediate (≤1 day), *n* (%)	122 (40.4)
Delayed (>1 day), *n* (%)	180 (59.6)
Rash, *n* (%)	296 (98.0)
Urticaria	71 (23.5)
Maculopapular	101 (33.4)
Unspecified rash	137 (45.4)
Other symptoms (fever, angioedema, vomiting, diarrhea, anaphylaxis, dyspnea)	18 (6)
Rash duration (days), Me (IQR)	1.0 (0–3)
Interval index challenge (months), Me (IQR)^a^	9 (2.3–25.5)

Me, median; IQR, interquartile range.

a Time from index reaction to allergy evaluation: interval in months between the date of the index suspected penicillin reaction and the date of the diagnostic drug provocation test in the allergy clinic.

**Figure 1:**
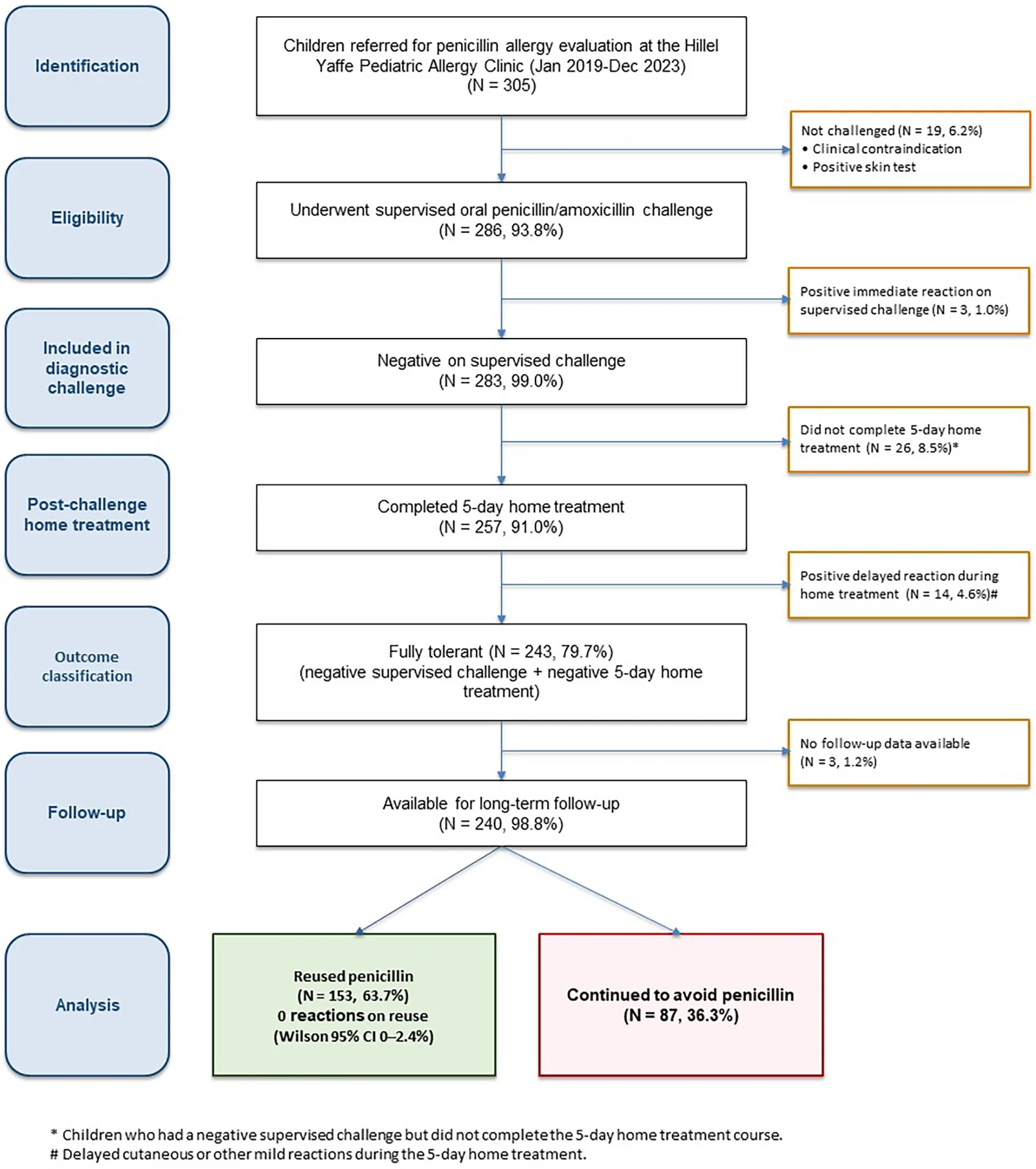
STROBE flow diagram of patients throughout the study.

Reported reactions were predominantly nonimmediate, with a median onset of 2 days after penicillin exposure. Immediate reactions, defined as occurring within 1–6 h after drug administration, were reported in 40.4% of patients, while 59.6% reported nonimmediate reactions. Cutaneous manifestations were reported in 98.0% of patients. Rash morphology was unspecified in 45.4% of cases, while 33.4% were described as maculopapular and 23.5% as urticarial. Other systemic symptoms, including fever, angioedema, vomiting, diarrhea, anaphylaxis, or dyspnea, were reported in 6% of patients. The median duration of rash was 1 day, ranging from 10 min to 120 days. The single case with the longest reported duration (120 days) was an urticarial reaction without mucosal, systemic, or other SCAR-defining features. Rash duration was recorded by caregiver report rather than direct clinical follow-up and may in some cases reflect residual skin change rather than ongoing active eruption. The median interval from index reaction to allergy evaluation was 9.0 months.

Challenge outcomes are described in Table 2. Skin prick test (SPT) was performed in 52.5% of patients, with positive results in 1.9% (*n* = 3). Intradermal test (IDT) was performed in 53.8%, with positive results in 4.9% (*n* = 8). 50/71 children (70.4%) with urticaria underwent skin testing, vs. 115/231 (49.8%) of children without urticaria (*p* = 0.004). Oral provocation testing was subsequently performed in 93.8% of the cohort and was well tolerated in 98.6% of challenged patients, with no reaction observed. Mild, non-life-threatening allergic reactions occurred in 1.05% of challenged patients (*n* = 3). No severe cutaneous adverse reactions, anaphylaxis requiring epinephrine, or hospitalizations occurred during supervised challenge.

**Table 2:** Challenge outcomes.

Variables	Values
SPT performed, *n* (%)	160 (52.5)
Positive	3 (1.9)
ID test performed, *n* (%)	164 (53.8)
Positive	8 (4.9)
Provocation test performed	286 (93.8)
Positive	3 (1.05)
Continued penicillin 5-day home treatment	257 (84.3)
Positive response (mild rash, vomiting)	14 (5.4)
Overall challenge not performed^a^	19 (6.2)
Negative tolerant	243 (79.7)
Negative clinic only/home not done	26 (8.5)
Not challenged (contraindicated/positive skin test)	19 (6.2)
Positive delayed (home)	14 (4.6)
Positive immediate	3 (1.0)
Any positive among challenged (primary outcome)	17 (5.94)
Any positive among total cohort	17 (5.57)

SPT, skin prick test; Me, median; IQR, interquartile range.

a Patients in whom neither supervised oral provocation nor the 5-day home treatment was performed.

Following a negative supervised challenge, 84.3% of patients completed the 5-day home course of penicillin as instructed. Mild adverse events during home treatment were reported in 14 patients (5.4%), consisting of cutaneous rash or vomiting. Full tolerance, defined as negative clinic provocation and negative 5-day home treatment, was documented in 79.7% of the cohort. Of the remainder, 8.5% had a negative clinic challenge but did not complete the home course, while 6.2% were not challenged because of a positive skin test or another clinical contraindication. The primary outcome, any positive reaction, occurred in 5.94% of challenged patients and in 5.57% of the entire cohort. No SCAR, anaphylaxis requiring epinephrine, or hospitalizations were reported during challenges.

The comparison between patients with a positive challenge and those who were fully tolerant is presented in Table 3. A trend toward older age was observed among patients with a positive challenge compared with tolerant patients (*p* = 0.069). Time to index reaction onset, rash duration, gender, indication for penicillin treatment, and the presence of urticaria and maculopapular rash were not significantly associated with challenge positivity. The only variable significantly associated with positivity was a longer interval between the index reaction and the challenge (*p* = 0.011). The indication for the original prescription was not a predictor of outcome, supporting a policy of direct challenge irrespective of indication.

**Table 3:** Predictors of positive challenge.

Variables	Negative tolerant (*n* = 243)	Positive (*n* = 17)	*P*-value
Age (years), Me (IQR)	3.5 (2.0–6.5)	6 (4.5–8)	0.069
Gender, Male, *n* (%)	124 (51.0)	11 (64.7)	0.322
Time (days), Me (IQR)	2 (1.0–4.0)	3 (1.0–7.0)	0.412
Rash duration (days), Me (IQR)	1.0 (0.0–3.0)	1.0 (0.0–3.0)	0.884
Time from index reaction to allergy evaluation (months), Me (IQR)	9.0 (2.0–24.0)	30.0 (8.0–68.0)	**0.011**
Treatment reason, *n* (%)			0.949
Acute otitis media	81 (33.3)	6 (35.3)	
Streptococcal pharyngitis	43 (17.7)	2 (11.8)	
Non-specific pharyngitis	24 (9.9)	2 (11.8)	
Pneumonia	27 (11.1)	2 (11.8)	
Other	8 (3.3)	0 (0.0)	
Diagnosis unclear	58 (23.9)	5 (29.4)	
Urticaria, *n* (%)	52 (21.4)	4 (23.5)	0.768
Maculopapular rash, *n* (%)	80 (32.9)	5 (29.4)	1.000

Me, median; IQR, interquartile range.

*P*-values were derived using Chi-square or Fisher's exact test as appropriate to assess the association between categorical variables. Comparison of continuous variables was analyzed by the Mann–Whitney test.

Bold values indicate statistically significant results (p < 0.05).

In an exploratory complete-case multivariable logistic regression analysis including 255 patients and 15 positive events, the events-per-variable ratio was 3.0. Therefore, adjusted estimates should be interpreted cautiously. A longer interval from the index reaction to allergy evaluation remained associated with challenge positivity (OR: 1.02 per month, 95% CI: 1.003–1.043, *p* = 0.022). Female sex showed a non-significant inverse association with positivity (OR: 0.31, 95% CI: 0.09–1.07, *p* = 0.064), while age, time to reaction onset, and urticaria were not significantly associated with positivity. Given the small number of positive events, these findings are exploratory and require validation in larger cohorts.

Table 4 presents long-term penicillin reuse and secondary outcomes. Among the 243 fully tolerant outcome patients, follow-up data on subsequent penicillin use were available for 98.8% of the patients. Of these, 63.7% (*n* = 153) reported subsequent penicillin reuse, whereas 36.3% (*n* = 87) had no documented subsequent penicillin use. A reason for non-use was documented for 17 of these 87 children (19.5%): 11 (12.6%) noted that no illness or clinical need for antibiotics arose during follow-up, and 6 (6.9%) noted persistent parental concern, miscommunication, or clinician hesitancy despite delabeling counselling; no reason was recorded for the remaining 70 children (80.5%). In contrast, among the 26 patients with a negative clinic challenge who did not complete home continuation, follow-up was available for 12; only 1 had subsequently reported penicillin reuse, suggesting that incomplete protocol completion may be associated with reduced subsequent caregiver or prescriber confidence in reintroducing penicillin. Given the very small sample size underlying this observation (follow-up available for only 12 of 26 patients, with a single reuse event), this finding should be interpreted as exploratory and hypothesis-generating rather than as a substantive conclusion. Notably, sustained tolerance was 100% among the 153 patients who reused penicillin after a negative challenge (Wilson 95% CI for reaction, 0–2.4%; i.e., tolerance 97.6%–100%), with no case of resensitization or severe reaction documented.

**Table 4:** Long-term penicillin reuse after negative challenge.

Variables	Values	*P* value
Fully tolerant patients, *n* (%)	243 (79.7)	–
Reuse data available follow up completed, *n* (%)	240 (98.8)	–
Reuse, *n* (%)	153 (63.7)	–
Avoided, *n* (%)	87 (36.3)	–
Reaction after reuse (sustained tolerance)	0 (0.0)	–
Time since provocation, reusers/avoiders, Me (IQR)	24.0 (0–24.0)/36 (24.0–72.0)	**0**.**0009**
Age, reusers/avoiders, Me (IQR)	3.0 (1.5–5.0)/5.3 (2.5–10.0)	**0**.**0007**

Me, median; IQR, interquartile range.

*P*-values were derived using Mann–Whitney test.

Bold values indicate statistically significant results (p < 0.05).

Factors associated with reuse were explored among all patients with a negative challenge and known reuse status. Patients who reused penicillin were significantly younger at the time of index reaction (*p* = 0.0007) and had a significantly shorter time since provocation (*p* = 0.0009). There was no significant association with gender (*p* = 0.59), rash morphology (urticaria *p* = 0.43, maculopapular *p* = 0.22) or the original indication (*p* = 0.283).

In multivariate logistic regression for predictors of reuse, including age, gender, time to reaction onset, and time since provocation (*n* = 175 with complete data, pseudo R^2^ = 0.234; likelihood-ratio *p* < 0.001), younger age was independently associated with higher odds of reuse (OR: 0.79 per additional year of age, 95% CI: 0.71–0.88, *p* < 0.001) and longer time since provocation was independently associated with lower odds of reuse (OR: 0.98 per month, 95% CI: 0.96–0.998, *p* = 0.014). Gender and time to index reaction were not significant predictors.

## 4 Discussion

In this large pediatric cohort referred for suspected penicillin allergy, true allergy was confirmed in only a small minority of children. The vast majority of patients tolerated a direct oral penicillin challenge without significant adverse reactions, further supporting the growing body of evidence that most penicillin allergy labels in children are inaccurate. These findings highlight a clinically important gap between establishing penicillin tolerance and translating that information into routine antibiotic use. Notably, reported reactions in our cohort were predominantly nonimmediate, mild, and cutaneous, consistent with prior studies indicating that such presentations are frequently associated with intercurrent viral infections rather than true immune-mediated hypersensitivity (1, 8).

The finding that a longer interval between the index reaction and challenge was the only variable significantly associated with challenge positivity does not have an obvious immunological explanation; there is no established mechanism by which delaying re-exposure would increase, rather than decrease, the likelihood of a true reaction. We consider it more likely that this association reflects referral or selection bias: children with a more concerning index history, or whose caregivers or referring physicians were more hesitant to pursue re-exposure, may have been referred for formal evaluation later, thereby enriching this subgroup for genuine reactors. This association should therefore be interpreted as hypothesis-generating rather than as evidence of a time-dependent biological mechanism.

Our findings align with previous literature demonstrating that the large majority of patients labeled as penicillin-allergic tolerate penicillin on formal evaluation (9, 10). The low rate of positive skin testing and the very low incidence of reactions during challenge in our cohort further support the limited diagnostic utility of skin testing in many pediatric patients, particularly those with low-risk histories. These observations reinforce the growing adoption of risk-stratification strategies, including validated tools such as PEN-FAST, and direct oral challenges without prior skin testing in selected populations (12, 18). A recent large meta-analysis confirmed that reactions to direct penicillin challenges are infrequent overall (3.5%) and even lower among children (2.2%) than adults, with severe reactions rare across age groups (9). No severe or life-threatening reactions occurred during challenge in our cohort, underscoring the safety of this approach in a controlled clinical setting and supporting the 2022 Joint Task Force practice parameter recommending direct challenge as first-line evaluation in low-risk children (12).

The association between a longer interval from the index reaction to allergy evaluation and challenge positivity should be interpreted cautiously. Although this association remained statistically significant after adjustment, there is no clear basis to infer that a longer interval itself increases the likelihood of persistent penicillin hypersensitivity. Because referral timing was not standardized, this finding may reflect selection or referral patterns, with children evaluated later potentially representing a subgroup with greater ongoing concern regarding the original reaction or other unmeasured characteristics. Given the small number of positive challenge events, this observation should therefore be considered hypothesis-generating and requires confirmation in prospective cohorts with standardized referral and evaluation intervals.

The number of positive challenge outcomes was small. Although 17 children had a positive outcome overall, the complete-case multivariable model included only 15 positive events across five predictor variables, corresponding to 3 events per variable. This low events-per-variable ratio increases the risk of unstable regression coefficients and wide confidence intervals. Accordingly, the adjusted associations, including the association between time to evaluation and positivity, should be regarded as exploratory and hypothesis-generating rather than definitive. In particular, the non-significant borderline association observed for female sex should not be interpreted as evidence of an independent sex-related effect.

Beyond diagnostic outcomes, our study provides important insights into post–delabeling antibiotic utilization in real-world settings. While nearly two-thirds of the patients subsequently received penicillin without adverse effects, a substantial proportion did not, despite documented tolerance. Although lack of clinical indication accounted for the majority of these cases, a meaningful subset avoided penicillin because of misunderstanding of medical recommendations or persistent concerns about potential reactions. This highlights a critical and under-recognized gap between successful diagnostic delabeling and its translation into routine clinical practice.

Our findings are broadly consistent with the limited pediatric literature examining antibiotic use after penicillin allergy delabeling, while also highlighting differences in study populations, follow-up duration, and definitions of antibiotic reuse (Table 5). In a 5-year follow-up study of children evaluated at a tertiary pediatric allergy center, Kitsos et al. reported that 76.4% of patients subsequently reused the previously suspected antibiotic, a proportion higher than the 63.7% observed in our cohort (21). This difference may reflect variations in the definition of reuse, follow-up methodology, patient characteristics, and the proportion of patients for whom subsequent antibiotic treatment was clinically indicated. In contrast, Vyles et al. reported documented penicillin-class prescribing in 36% of children approximately 1 year after delabeling, while Pinto et al. reported willingness to accept penicillin in 86% of participants at 1-year follow-up (20, 26). Taken together, these studies support the feasibility and apparent durability of penicillin delabeling while demonstrating that successful diagnostic delabeling does not uniformly result in subsequent real-world penicillin use. Our study extends this literature by providing long-term follow-up of up to 5 years in a relatively large pediatric cohort and by specifically examining actual reported penicillin reuse and continued avoidance after documented tolerance.

**Table 5:** Comparison with prior long-term follow-up studies of pediatric penicillin allergy delabeling.

Study	Country/setting	Pediatric population	N analyzed^a^	Max. follow-up	Subsequent penicillin use (definition)	Reported reactions on reuse
Present study (Schichter-Konfino et al.)	Israel tertiary allergy clinic	Yes—median age 4 y	240	Up to 5 years	63.7% (153/240) reused penicillin after negative challenge ± home treatment	no case of resensitization or severe reaction documented
Kitsos et al., 2026 (21)	Greece tertiary pediatric allergy centre	Yes—referred 2017–2020	106	5 years	76.4% (81/106) reused the previously suspected antibiotic at least once	no severe reactions or hospitalizations. Resensitization could not be completely excluded because retesting was not performed.
Vyles et al., 2018 (26)	USA pediatric emergency department	Yes—low-risk symptoms	100	∼1 year	36% (36/100) received ≥1 documented penicillin-class prescription^b^	1/36 (2.8%) developed rash → relabeled; no severe reaction
Pinto et al., 2023 (20)	Australia tertiary hospital, community follow-up	No—adult cohort	59 (of 86 delabeled; 27 lost to follow-up, 2 deceased, 25 uncontactable)	1 year	47.0% (28/59) received a penicillin-based antibiotic between delabeling and follow-up (PCP records + patient interview)^c^; 72.9% (43/59) said they would accept a future penicillin prescription, 13.6% (8/59) said they would not	Not reported for post-delabeling reuse; during the original 3-day in-hospital delabeling challenge, 8/86 (9.3%) had GI upset and 2/86 (2.3%) had a delayed benign rash
Lachover-Roth et al., 2019 (27)	Israel tertiary allergy unit, ambulatory	No—mixed adult/pediatric (447/654, 68% < 18 y) ambulatory cohort	639	Up to ∼5.3 years (median ∼3.1 y; range ∼0.6–5.3 y)	70.0% (447/639) used penicillin ≥ 1 time after negative OCT (phone survey and/or pharmacy purchase record); 51.4% (336/654) still carried a penicillin allergy label in the EMR despite a negative OCT	4.6% reported a suspected reaction on reuse, mostly benign/delayed rash; no severe or anaphylactic reactions

a N analyzed refers to the denominator used for the reported reuse/use percentage in each study (delabeled patients with follow-up data), not the full referred cohort.

b Vyles et al. report the percentage of delabeled patients with a documented penicillin-class prescription filled during follow-up, which is not equivalent to a patient-reported decision to reuse or avoid penicillin; figures are not directly comparable to studies reporting reuse as a patient/caregiver-level outcome.

c Pinto et al. report both actual receipt of penicillin-based antibiotics (47%, 28/59, via Primary Care Practitioner records and patient interview) and self-reported willingness among the 59 of 86 delabeled patients successfully interviewed at ≥1-year follow-up.

These findings are consistent with previous studies demonstrating that inappropriate persistence of penicillin allergy labels or failure to act on their removal remains a significant barrier to optimal antibiotic use (26, 27). Behavioral factors, including parental anxiety, miscommunication, and physician prescribing habits, likely contribute to this phenomenon. System-level barriers such as incomplete documentation in EMR and limited communication between specialists and primary care providers may further limit the impact of delabeling interventions.

From a clinical and public health perspective, our results emphasize that penicillin allergy evaluation should be considered as an integral component of antimicrobial stewardship programs. Effective delabeling can improve antibiotic selection, reduce unnecessary use of broad-spectrum agents, and decrease healthcare costs. Realizing these benefits fully, however, requires strategies that extend beyond diagnostic testing to include clear communication with families, standardized documentation of results, and education of both caregivers and healthcare providers, an approach increasingly supported by studies successfully extending direct oral challenge programs into primary care and emergency department settings (11, 16, 17).

Interestingly, the rate of resensitization in our cohort was negligible, consistent with prior reports suggesting that true resensitization following a negative challenge is rare. A recent 5-year follow-up study of delabeled children similarly found that more than three-quarters safely reused the previously avoided antibiotic with no clinically significant reactions, and short-term follow-up data from the PALACE trial likewise demonstrated low rates of relabeling after direct challenge (21, 28). These findings further support the long-term safety of penicillin reintroduction after appropriate evaluation. Nevertheless, continued vigilance and appropriate counseling of families remain important, particularly in cases with atypical presentations.

Several limitations should be acknowledged. First, the retrospective design introduces potential selection bias and incomplete data capture, particularly regarding reaction history and long-term outcomes. Second, follow-up data were based on caregiver report, which may be subject to recall bias. Third, the absence of a standardized follow-up protocol and the lack of objective verification of subsequent antibiotic use (through pharmacy or prescription records) may further limit the precision of these findings. Additionally, this study did not systematically ascertain whether children with no documented subsequent penicillin use had a clinical indication for antibiotic therapy during follow-up; absence of documented use may therefore reflect absence of a clinical opportunity, incomplete communication, or persistent caregiver concern rather than deliberate avoidance. This limitation is compounded by the relatively short follow-up available in our cohort: the median interval from index reaction to allergy evaluation was 9.0 months, and in a pediatric population that may require antibiotics only once every 1–2 years, this window may be too brief to reliably capture a subsequent re-exposure event. Consistent with this concern, a reason for non-use could not be ascertained for 70 of the 87 children (80.5%) who did not reuse penicillin, so it is not possible to distinguish genuine, persistent avoidance from the simple absence of a clinical indication for antibiotics during the observation period. A longer, ideally multi-year, follow-up period with structured ascertainment of antibiotic prescribing, as has been achieved in some prior cohorts, would be required to more definitively characterize post-delabeling penicillin reuse. In addition, the 5-day home continuation duration reflected institutional protocol rather than a prospectively tested comparison of exposure durations, and whether a shorter course would achieve comparable diagnostic yield remains untested; administering antibiotics without a confirmed infectious indication may also expose children to unnecessary adverse effects and contributes to unnecessary antimicrobial exposure, a consideration relevant to antimicrobial stewardship. In addition, our study was not designed to compare different durations of drug provocation, and our findings do not establish that a 5-day challenge is necessary or superior to a shorter regimen; the duration reflected the clinical protocol used at our center during the study period rather than a prospectively evaluated comparison of challenge strategies. Administration of antibiotics for diagnostic purposes in the absence of an infectious indication represents additional antimicrobial exposure and may carry a risk of adverse effects as well as broader antimicrobial stewardship concerns, including antimicrobial resistance; the potential diagnostic benefit of detecting delayed reactions must therefore be balanced against unnecessary antibiotic exposure. Whether a shorter extended challenge would provide comparable diagnostic sensitivity with less antimicrobial exposure cannot be determined from our data and should be addressed in prospective comparative studies. Fourth, the follow-up questionnaire did not systematically record subsequent antibiotic prescriptions among children who did not reuse penicillin. Consequently, it was not possible to determine whether penicillin avoidance resulted in the use of alternative or broader-spectrum antibiotics. Future prospective studies should incorporate structured EMR and prescription-level data. Although patients were provided with written documentation for their treating physicians following a successful challenge, we did not have access to their subsequent electronic medical records and therefore could not verify whether this letter resulted in formal removal of the label from EMR. Future prospective studies should systematically assess the persistence or removal of allergy labels following negative challenge. Finally, the small number of challenge-positive events limits the reliability of analyses attempting to identify predictors of challenge positivity. Despite these limitations, the relatively large pediatric cohort covering the full spectrum of low-risk phenotypes seen in real-world clinical setting, the use of a standardized protocol including 5-day home continuation which captures delayed T-cell reactions and high follow-up completion rate, including follow-up periods of up to 5 years in a subset of patients, providing long-term data on reuse patterns rarely reported in children, enhance the external validity and generalizability of our results.

The 5-day home continuation used in our cohort was part of the clinical diagnostic protocol routinely employed at our center during the study period. Following a negative supervised graded oral challenge on the first day, patients were instructed to continue penicillin at home for 5 days in order to detect non-immediate hypersensitivity reactions that might not become apparent during the initial supervised observation period. Importantly, no severe adverse events associated with the 5-day home exposure were reported during follow-up. Fourteen children (5.4%) developed a mild adverse event during the extended course, consisting of cutaneous rash or vomiting; these reactions were mild and may represent delayed hypersensitivity reactions, which were specifically the type of reaction the extended challenge protocol was intended to identify. Thus, the extended home exposure provided additional diagnostic information beyond the immediate supervised challenge.

The subgroup of children who did not complete the 5-day home continuation also warrants cautious interpretation. Only 12 of the 26 patients in this subgroup had available follow-up, and only one subsequently reported penicillin reuse. Although this pattern may be consistent with a possible role of incomplete protocol completion in subsequent antibiotic avoidance, the very small sample size prevents meaningful inference. This observation should therefore be considered hypothesis-generating and warrants prospective evaluation.

Our findings confirm that penicillin allergy is overdiagnosed in children and that direct oral challenge is a safe and effective strategy for delabeling in this large pediatric cohort. Direct oral challenge was safe and successfully established penicillin tolerance in the large majority of children. These results reinforce that most children labeled as penicillin-allergic are not truly allergic and can be effectively delabeled through a direct oral challenge. However, successful delabeling did not consistently translate into penicillin reuse despite negative testing; more than a third of patients did not subsequently receive penicillin. Addressing these behavioral, educational, and system-level barriers is essential to bridge the gap between diagnostic delabeling and its clinical benefit, and to maximize the impact of penicillin allergy evaluation on antimicrobial stewardship in pediatric populations.

## Data Availability

The raw data supporting the conclusions of this article will be made available by the authors, without undue reservation.
